# From Epistaxis to a Platelet Count of Four: A Rare Presentation of Teicoplanin-Induced Thrombocytopenia

**DOI:** 10.7759/cureus.104648

**Published:** 2026-03-04

**Authors:** Abhishek Shrestha, Urvashi Jain, Ashby Mathew, Kenny Ha

**Affiliations:** 1 Internal Medicine, Princess Alexandra NHS Trust, Harlow, GBR; 2 Geriatrics, Princess Alexandra NHS Trust, Harlow, GBR

**Keywords:** adverse drug event, drug-induced immune thrombocytopenia, g i bleeding, glycopeptide, nasal bleed, osteo-myelitis, platelets count, teicoplanin, thrombocytopenia

## Abstract

Teicoplanin is a glycopeptide antibiotic used for serious Gram-positive infections, particularly those caused by methicillin-resistant Staphylococcus aureus (MRSA) and resistant Enterococcus species. While generally safe, it may rarely lead to haematological complications such as drug-induced immune thrombocytopenia (DITP), a potentially life-threatening disorder caused by immune-mediated platelet destruction. We report a case of severe symptomatic thrombocytopenia occurring 10 days after starting teicoplanin therapy in an elderly patient with a diabetic foot infection. Comprehensive clinical and laboratory assessments excluded alternative causes. Teicoplanin was stopped, and the patient received a single dose of intravenous immunoglobulin (IVIG), which led to gradual platelet recovery and cessation of bleeding. This report underscores the importance of routine blood count monitoring, early recognition of thrombocytopenia, and timely discontinuation of teicoplanin to prevent major bleeding. It also highlights the necessity of further studies to clarify the mechanisms of teicoplanin-induced thrombocytopenia (TIT), improve drug-specific antibody testing, and define the role of IVIG in treatment.

## Introduction

The emergence and global spread of multidrug-resistant Gram-positive pathogens, particularly methicillin-resistant Staphylococcus aureus (MRSA) and resistant Enterococcus species, have led to increased reliance on glycopeptide antibiotics [1]. Teicoplanin is a semisynthetic glycopeptide that inhibits bacterial cell wall synthesis by binding to the D-ala-D-ala terminus of peptidoglycan precursors [2]. It is usually reserved for the treatment of serious infections caused by β-lactam-resistant Gram-positive bacteria, including osteomyelitis, septic arthritis, and endocarditis [3]. Teicoplanin is often preferred due to its longer half-life, once-daily dosing, lower incidence of nephrotoxicity, availability of intramuscular administration, and suitability for outpatient therapy [4]. Common adverse drug reactions include fever, allergic reactions, thrombophlebitis, mild transaminitis, and reversible leukopenia [4]. Although thrombocytopenia is a rare adverse effect, severe symptomatic cases are even less common [5].

Drug-induced immune thrombocytopenia (DITP) is an immune-mediated disorder characterised by a sudden and often severe reduction in platelet count, frequently dropping below 20 × 10⁹/L [5,6]. Severe thrombocytopenia, particularly when platelet counts fall below 20 × 10⁹/L, is clinically significant because it substantially increases the risk of spontaneous bleeding, including mucosal haemorrhage and potentially life-threatening intracranial haemorrhage. Teicoplanin-induced thrombocytopenia (TIT) is a specific, sometimes under-recognised, form of DITP associated with the glycopeptide antibiotic teicoplanin, typically presenting with a slightly delayed but clinically important decline in platelet counts [6]. Diagnostic evaluation can be challenging due to factors such as polypharmacy, comorbidities, and limited availability of confirmatory laboratory tests [5,6]. This case report describes a rare instance of symptomatic, severe teicoplanin-induced immune thrombocytopenia, emphasising the diagnostic challenges and approaches to management.

## Case presentation

**Figure 1:**
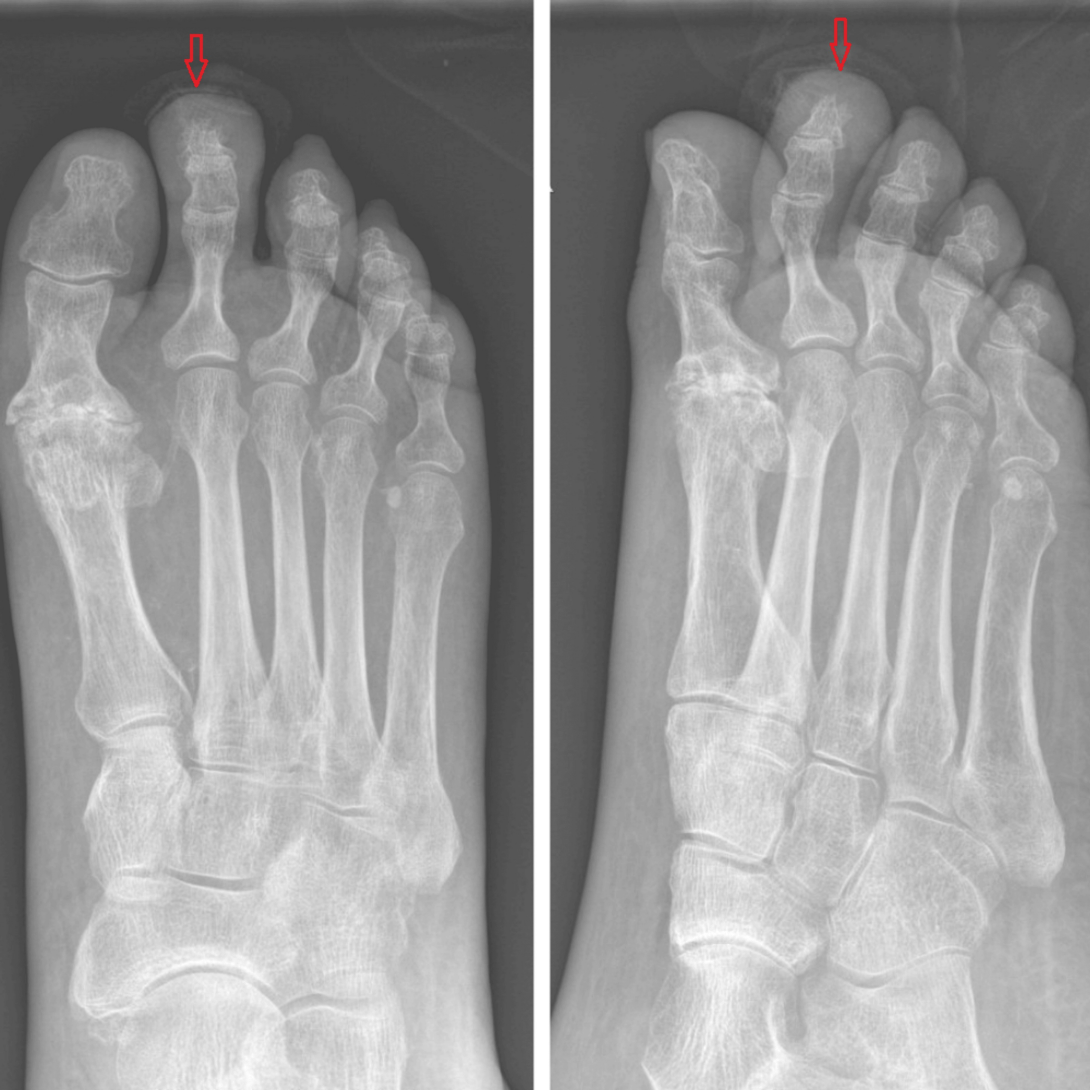
Right-foot X-ray - AP and lateral views Xray Right foot showed irregularity of the terminal tuft of the distal phalanx of second toe with several cortical erosions; significant surrounding soft tissue swelling noted, findings suggestive of osteomyelitis.

An 85-year-old female presented to the hospital after experiencing an episode of epistaxis and a two-day history of black, loose stools consistent with suspected melena. Routine blood investigations conducted by community district nurses revealed severe thrombocytopenia (platelet count: 4 × 10⁹/L). Ten days prior, the patient had been admitted with a right second toe diabetic foot infection. Wound culture identified Staphylococcus aureus, and plain radiography indicated osteomyelitis of the second toe (Figure 1). Her medical history included type 2 diabetes mellitus, stage 4 chronic kidney disease, atrial fibrillation, and previous deep vein thrombosis. Her regular medications included doxazosin, furosemide, Humalog insulin, omeprazole, and warfarin. She had a documented allergy to septrin.

Teicoplanin was commenced for moderate diabetic foot osteomyelitis, with concern for MRSA following consultation with the microbiology team. The patient received teicoplanin 600 mg every 12 hours for three loading doses in accordance with local guidelines. She was subsequently discharged on intravenous teicoplanin 600 mg every three days, adjusted for renal impairment (creatinine clearance: 15 mL/min), with a planned six-week course. The treatment was administered through the Hospital at Home (virtual ward) service. Her pre‑dose (trough) teicoplanin level remained within the normal therapeutic range.

On admission, repeat blood tests confirmed a platelet count of 4 × 10⁹/L (Table 1). She had no prior history of thrombocytopenia, and there had been no recent exposure to heparin. Her platelet count 10 days earlier, during her previous admission, had been 155 × 10⁹/L. Clinical examination demonstrated stable vital signs and unremarkable systemic findings, except for mild purpuric rashes on both lower legs. The diabetic foot ulcer exhibited no active discharge or soakage. On per rectal examination, no melena was noted.

**Table 1:** Complete blood count Table 1 shows the components of a full blood count panel, including a platelet count of 4 x 10^9^/L, on day 11 of teicoplanin treatment for diabetic foot osteomyelitis. At this time, the patient presented to the A&E with black-colored stool and an episode of epistaxis. Hb: haemoglobin; MCV: mean corpuscular volume; MCH: mean corpuscular haemoglobin

Blood parameters	Results	Reference range
Hb (g/L)	108	115 - 165
WBC (x10^9^/L)	5.3	4.0 - 11.0
Platelets (x10^9^/L)	4	150 - 450
MCV (fL)	95	76 - 96
RBC (x10^12^/L)	3.58	3.80 - 4.80
MCH (pg)	30.2	27 - 32
Neutrophils (x10^9^/L)	4.3	2.0 - 8.0
Lymphocytes (x10^9^/L)	0.6	1.0 - 4.8
Monocytes (x10^9^/L)	0.3	0.2 - 0.8
Eosinophils (x10^9^/L)	0.1	0.0 - 0.4

Laboratory investigations indicated mild chronic anaemia (Hb: 108 g/L), with otherwise normal biochemistry and coagulation profiles. The patient's renal function was consistent with stage 4 chronic kidney disease and had remained stable over the past year, with no evidence of acute deterioration or drug-related nephrotoxicity. Her INR remained within the therapeutic range, and warfarin‑associated bleeding was therefore excluded as a contributing factor. Teicoplanin was discontinued due to suspected drug-induced thrombocytopenia (DTP). Warfarin was withheld because of the increased bleeding risk. No additional episodes of black stools occurred during admission; hence, melena was excluded based on clinical findings and stable hemoglobin results.

The case was reviewed with the haematology team, who recommended viral and autoimmune screening. Platelet transfusion was not advised in the absence of active bleeding on review. Steroids were contraindicated because of the ongoing diabetic foot infection. The patient received a single dose of intravenous immunoglobulin (IVIG) in view of a profoundly low platelet count and the associated risk of further bleeding. Daily blood counts were monitored.

Based on microbiology recommendations, oral flucloxacillin was initiated for six weeks due to stable inflammatory markers, absence of sepsis, and overall clinical stability. Serial blood tests demonstrated gradual improvement in platelet counts. Viral serologies for hepatitis A, B, C, E, CMV, EBV, and HIV were negative. Lactate dehydrogenase (LDH), haptoglobin, blood film, antinuclear antibody (ANA), rheumatoid factor (RF), immunoglobulin levels, serum electrophoresis, anticardiolipin antibodies, and hematinics (vitamin B12, folate, ferritin, iron) were all within normal limits.

After one week, platelet counts increased to 68 × 10⁹/L. In the absence of further bleeding and with improving counts, the patient was discharged with outpatient follow-up arranged with haematology, diabetic foot, and podiatry services. She was advised to avoid future exposure to glycopeptide antibiotics (including teicoplanin and vancomycin) because of the risk of recurrent thrombocytopenia, and this alert was added to her electronic medical record. Repeat blood tests performed three weeks later as an outpatient showed a further increase in platelet levels to 132 × 10⁹/L.

## Discussion

DTP is a rare but serious adverse drug reaction that carries a high risk of haemorrhage [6]. DTP can be broadly classified into two groups based on the underlying mechanism. Non-immune-mediated thrombocytopenia results from the direct cytotoxic effects of the drug molecule on platelets or megakaryocytes [6,7]. In contrast, immune-mediated thrombocytopenia involves the formation of antibodies that bind platelet-specific glycoprotein complexes in the presence of the offending drug [7]. DITP is characterised by at least six distinct mechanisms through which drug-induced antibodies mediate platelet destruction. These mechanisms comprise drug-dependent antibody reactions (e.g., quinine), hapten-dependent antibody reactions (e.g., penicillin), fiban-induced thrombocytopenia (e.g., eptifibatide), drug-specific antibody reactions (e.g., abciximab), induction of autoantibodies (e.g., L-dopa), and immune complex-mediated reactions (e.g., heparin) [6,8].

Among the proposed mechanisms, the drug-dependent antibody (DDabs) mechanism is the most common cause of DITP. In this process, antibodies bind to platelet glycoproteins, most frequently GPIIb/IIIa or GPIb/IX, resulting in rapid platelet clearance by the reticuloendothelial system. This mechanism of platelet destruction was first described with quinine, an antimalarial drug. Currently, more than 100 medications have been implicated in this category, including teicoplanin [5]. Teicoplanin-dependent antibodies have been demonstrated in thrombocytopenic patients, with GPIIb/IIIa identified as a primary target antigen [9]. Interestingly, these antibodies have also been detected in some patients with normal platelet counts, suggesting that antibody presence alone may not be sufficient to cause clinically significant thrombocytopenia [9].

A recent retrospective observational study found that TIT occurred in approximately 4-5% of patients, as assessed by the Adverse Drug Reaction Probability Scale (Naranjo scale). Of 482 patients, 22 developed thrombocytopenia with platelet counts below 100 × 10³/µL. However, only two individuals experienced bleeding manifestations, corresponding to a symptomatic thrombocytopenia incidence of 0.41%, which is notably rare. [4]. This aligns with the infrequency of severe symptomatic presentations such as the one described in our case. The study also concluded that age, renal function, and teicoplanin dose were not associated with a higher risk of TIT development [4].

The diagnosis of DITP is supported by the onset of thrombocytopenia after exposure to a suspected drug, complete and sustained platelet recovery after discontinuation, exclusion of other causes, and recurrence upon re-exposure. Confirmation requires either a drug challenge or demonstration of DDAbs in vitro [6]. Laboratory tests for antibodies that bind to platelets in the presence of drugs or their metabolites are available. However, these assays offer limited clinical benefit because they are not automated, are time-consuming, require significant technical expertise, and are not widely available. As a result, diagnosis is often based on clinical features without laboratory confirmation [5,6,8]. The clinical manifestations of DITP are variable, ranging from asymptomatic laboratory abnormalities to mucosal bleeding, such as purpura or epistaxis. In rare cases, patients may experience severe complications, including gastrointestinal, pulmonary, or intracranial haemorrhage [5,8]. In this case, the patient was admitted with melena, epistaxis, and a purpuric rash on both lower extremities.

Management of DITP primarily involves prompt discontinuation of the suspected drug. Platelet counts typically begin to recover after approximately four to five half-lives of the drug or its active metabolites. Platelet transfusions are commonly administered to patients with severe thrombocytopenia who develop wet purpura, given the association of this clinical sign with an increased risk of intracranial haemorrhage. However, the therapeutic efficacy of platelet transfusion in this context has not been formally evaluated [8]. Moreover, platelet transfusions remain controversial and are generally considered ineffective while the drug or its metabolites persist in circulation [6]. IVIG may be considered in cases of severe thrombocytopenia with active bleeding or in patients at high risk of bleeding, although evidence supporting its routine use is limited [5,6,7].

Our patient presented with severe symptomatic thrombocytopenia secondary to teicoplanin administration. Evaluation using the Naranjo Scale suggested a probable adverse drug reaction, prompting immediate discontinuation of teicoplanin [10]. After administration of a single dose of IVIG, platelet counts improved, and no further bleeding occurred. Although differential diagnoses were excluded through clinical examination, laboratory investigations, and imaging, specific antibody testing was not performed; therefore, the diagnosis was established based on clinical assessment.

## Conclusions

TIT is a rare but potentially life-threatening adverse drug reaction that may be easily overlooked. This report emphasises the importance of baseline and interval full blood count monitoring, including platelet counts, in patients receiving teicoplanin therapy, particularly those managed in virtual ward settings, especially in the elderly population. Early identification of thrombocytopenia and prompt discontinuation of the offending agent are crucial to prevent progression to severe bleeding complications. Moreover, this report highlights the need for further research to better elucidate the pathophysiology of TIT, to develop reliable drug-specific antibody assays, and to establish the role of IVIG in its management.
